# Metabolic Reprogramming of Mammary Epithelial Cells during TGF-β-Induced Epithelial-to-Mesenchymal Transition

**DOI:** 10.3390/metabo11090626

**Published:** 2021-09-15

**Authors:** Wan Hua, Sarantos Kostidis, Oleg Mayboroda, Martin Giera, Marten Hornsveld, Peter ten Dijke

**Affiliations:** 1 Oncode Institute and Cell Chemical Biology, Leiden University Medical Center, 2300 RC Leiden, The Netherlands; huawan825@outlook.com (W.H.); M.Hornsveld@lumc.nl (M.H.); 2 Institute of Rare Diseases, West China Hospital, Sichuan University, Chengdu 610041, China; 3 Center for Proteomics and Metabolomics, Leiden University Medical Center, 2333 ZA Leiden, The Netherlands; s.kostidis@lumc.nl (S.K.); O.A.Mayboroda@lumc.nl (O.M.); m.a.giera@lumc.nl (M.G.)

**Keywords:** NMuMG breast cells, TGF-β, epithelial-to-mesenchymal transition (EMT), metabolism, choline kinase α (CHKα)

## Abstract

The cytokine transforming growth factor-β (TGF-β) can induce normal breast epithelial cells to take on a mesenchymal phenotype, termed epithelial-to-mesenchymal transition (EMT). While the transcriptional and proteomic changes during TGF-β-induced EMT have been described, the metabolic rewiring that occurs in epithelial cells undergoing EMT is not well understood. Here, we quantitively analyzed the TGF-β-induced metabolic reprogramming during EMT of non-transformed NMuMG mouse mammary gland epithelial cells using nuclear magnetic resonance (NMR) spectroscopy. We found that TGF-β elevates glycolytic and tricarboxylic acid (TCA)-cycle activity and increases glutaminolysis. Additionally, TGF-β affects the hexosamine pathway, arginine-proline metabolism, the cellular redox state, and strongly affects choline metabolism during EMT. TGF-β was found to induce phosphocholine production. A kinase inhibitor RSM-93A that inhibits choline kinase α (CHKα) mitigated TGF-β-induced changes associated with EMT, i.e., increased filamentous (F)-actin stress fiber formation and N-Cadherin mesenchymal marker expression.

## 1. Introduction

Epithelial-to-mesenchymal transition (EMT) is a dynamic process in which cuboidal epithelial apical–basal polarized cells convert into motile cells with a mesenchymal spindle-shaped phenotype. This transition is accompanied by a decrease in expression of epithelial markers such as E-cadherin and increased expression of mesenchymal markers such as N-cadherin and Vimentin [1,2]. EMT is a key process for embryonic and organ development. Primitive epithelial cells transition into motile mesenchymal cells at various stages of embryogenesis, and mesenchymal cells produced by EMT can transition back into epithelial cells by undergoing mesenchymal-to-epithelial transition (MET) [1]. In the adult body, EMT is associated with wound healing, organ fibrosis, and cancer progression [3]. EMT can facilitate cancer cell migration out of primary tumors, leading to invasion into distant tissues. Moreover, EMT can provide cancer cells with characteristics of stem cells and thereby increases the resistance to cancer therapy [4,5].

The cytokine transforming growth factor-β (TGF-β) is a major driver of EMT. TGF-β is a multifunctional cytokine that exerts a wide range of context-dependent biological responses. It is of pivotal importance during embryonic epithelial organogenesis and maintenance of adult tissue homeostasis [6,7,8,9]. Perturbation of TGF-β signaling has been linked to a multitude of developmental disorders and pathologies, including fibrosis and cancer. TGF-β signaling plays a dual role in cancer progression [10]. In early tumorigenesis, TGF-β acts as a tumor suppressor by repression of proliferation and stimulation of apoptosis in normal and premalignant epithelial cells [11,12]. At later stages of tumor progression, cancer cells become insensitive to TGF-β-induced cytostatic responses as a result of genetic alteration of the pathway through mutations. At that stage, TGF-β can act as a tumor promoter by acting directly on cancer cells, stimulating metastasis-associated EMT [13,14,15].

Recent findings have suggested that EMT-associated metabolic changes can play a role in this process [16,17]. However, the many metabolic changes that occur in cells that undergo EMT in response to TGF-β, and their functional role therein, remain to be fully understood. Increasing evidence in different cancers indicates that TGF-β promotes glycolysis while inducing EMT and upregulates the expression of several glycolytic metabolic enzymes, such as glucose transporter 1 (GLUT1), hexokinase 2 (HK2), 6-phosphofructo-2-kinase/fructose-2,6-biphosphatase 3 (PFKFB3), and pyruvate kinase M2 (PKM2) [18,19,20,21]. The mutation of certain genes encoding enzymes involved in the tricarboxylic acid (TCA) cycle, such as fumarate hydratase (*FH*), succinate dehydrogenase (*SDH*), and isocitrate dehydrogenase (*IDH*), have been directly associated with EMT [22,23,24,25]. However, these effects of TGF-β on cellular metabolism were observed in different model systems. Thus, a comprehensive characterization of TGF-β-induced metabolic wiring during EMT is lacking.

To obtain a better understanding of the role TGF-β plays in metabolic programming, we analyzed early TGF-β-induced metabolic changes of non-transformed immortalized breast NMuMG cells with state-of-the-art quantitative nuclear magnetic resonance (NMR) spectroscopy-based metabolomics [26]. We found that TGF-β triggers extensive metabolic reprogramming in NMuMG cells undergoing EMT. Among the described changes, three key metabolic pathways involving cytidine diphosphate choline (CDP-Cho) and biosynthesis of polyamines and proline were found to be regulated by TGF-β. TGF-β-induced phosphocholine production and a kinase inhibitor RSM-93A that inhibits choline kinase α (CHKα) (among other kinases) mitigated TGF-β-induced changes associated with EMT, i.e., increased filamentous (F)-actin stress fiber formation and N-Cadherin mesenchymal marker expression. Our results are consistent with the idea that TGF-β-dependent metabolic changes as necessary for EMT.

## 2. Results

### 2.1. TGF-β Induces EMT in NMuMG Cells

**Figure 1:**
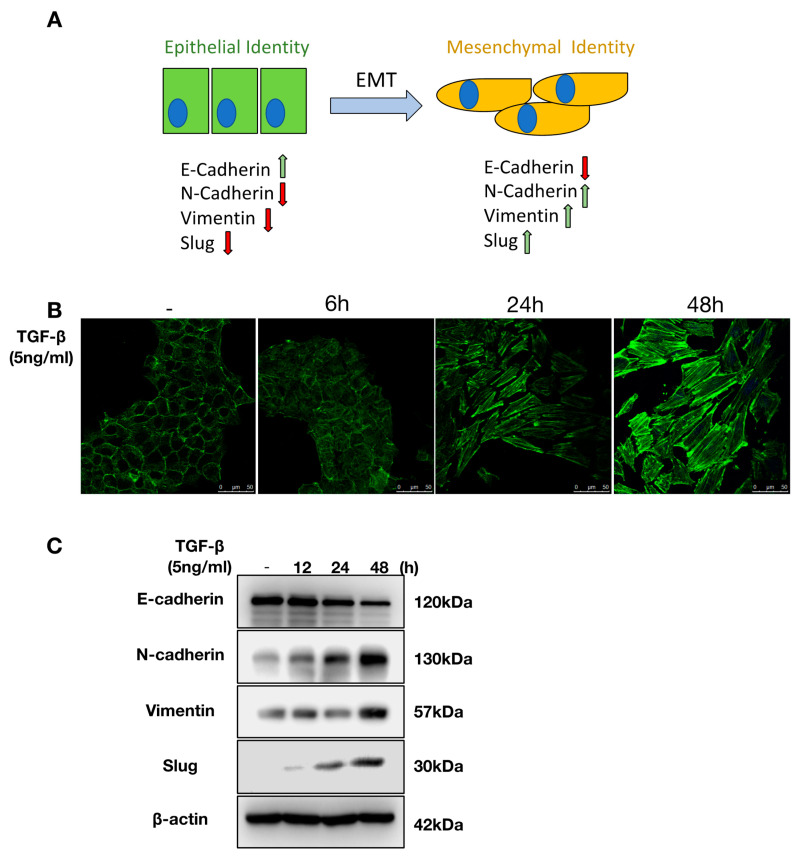
Transforming growth factor-β (TGF-β) stimulates epithelial-to-mesenchymal transition (EMT) in NMuMG cells. (**A**) Illustration of EMT in which epithelial cells switch morphologically into a mesenchymal phenotype. EMT is characterized by increased mesenchymal markers and decreased epithelial markers expression. (**B**) In response to TGF-β (5 ng/mL) stimulation, NMuMG cells acquired elongated F-actin stress fibers. (**C**) Upon TGF-β stimulation, the expression of E-cadherin is decreased, and expression of N-cadherin, Vimentin, and Slug is increased.

EMT is a biological process in which epithelial cells lose their apical–basal polarity and epithelial intercellular junctions to convert into a spindle-shaped fibroblast-like mesenchymal phenotype with increased mobility (Figure 1A). To test if TGF-β induces EMT of NMuMG cells, we stimulated these cells with TGF-β for 48 h. In untreated cells, phalloidin staining that measures filamentous (F-) actin organization showed a pericellular plasma membrane distribution of F-actin. In response to TGF-β stimulation, NMuMG cells acquired elongated F-actin stress fibers and changed their morphology from an epithelial to a spindle-shaped fibroblast-like mesenchymal phenotype (Figure 1B and Figure S1). Furthermore, upon TGF-β treatment, the expression of mesenchymal-associated protein markers N-cadherin and Vimentin increased, and epithelial marker E-cadherin decreased (Figure 1C and Figure S1). In addition, the EMT-inducing transcription factor Slug was also upregulated following TGF-β treatment (Figure 1C) [27]. Taken together, these results show a robust TGF-β-mediated induction of EMT in NMuMG over the course of 48 h.

### 2.2. TGF-β Triggers Metabolic Rewiring in NMuMG Cells

**Figure 2:**
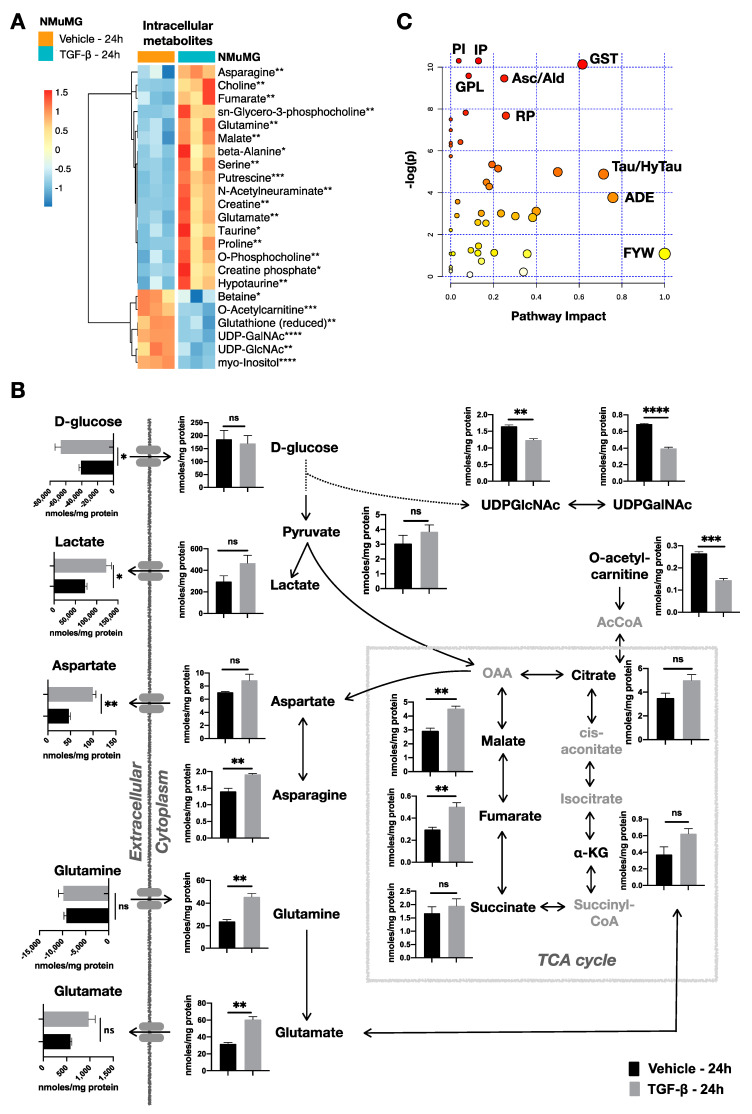
Analysis of TGF-β-induced metabolic changes in NMuMG cells after 24 h treatment. (**A**) Heatmap of standardized concentrations (z-scores) of significantly different (*p* < 0.05, n = 3) intracellular metabolites after addition of TGF-β (5 ng/mL) compared to vehicle solvent control. (**B**) Schematic representation of metabolic changes induced by TGF-β (5 ng/mL) in NMuMG cells, including glycolysis, tricarboxylic acid (TCA) cycle, and aspartate production. Results are shown as mean ± s.d; * *p* < 0.05; ** *p* < 0.01; *** *p* < 0.001; **** *p* < 0.0001; ns: non-significant. (**C**) Changes in metabolic pathways after TGF-β (5 ng/mL) or vehicle control treatment of NMuMG cells. Pathway scores were calculated based on the number of NMuMG cell-derived metabolites present in a pathway and the sign of change after TGF-β addition. PS of 1, as shown here for the pathway of choline metabolism in cancer, indicates that all detected metabolites in the pathway exhibited the same change after TGF-β addition. The thickness of each point represents the number of detected metabolites in each pathway. IP: inositol phosphate; PI: phosphatidylinositol; GST: glycine, serine, and threonine; RP: arginine and proline; Tau/HyTau: taurine and hypotaurine; ADE: alanine, aspartate, and glutamate; GPL: glycerophospholipid.

To examine whether the TGF-β-induced EMT is accompanied by metabolic changes, we performed quantitative metabolomics analysis of NMuMG treated with TGF-β (or vehicle control) for 12 h, 24 h, and 48 h (Supporting Information Figure S2). Indeed, with EMT advancing after 24 h, we observed a metabolic reprogramming. Compared with untreated NMuMG cells, 23 intracellular metabolites were significantly changed (false discovery rate (FDR) adjusted *p* value; q < 0.05), 17 of which were more abundant and 6 were decreased after TGF-β treatment (Figure 2A). Numerous studies suggest the importance of glycolysis in EMT [28]. Here, we confirmed that TGF-β treatment induced NMuMG to consume significantly more glucose and produce and excrete more lactate (Figure 2B). We further observed significant differences in the products of the hexosamine pathway, a branch of intracellular glucose catabolism, in particular, UDP-GlcNAc and UDP-GalNAc (Figure 2B). These metabolic findings were further supported by analysis of the expression of genes encoding enzymes involved in glycolytic pathways. Quantitative real-time (qRT)-polymerase chain reaction (PCR) analysis of TGF-β-treated NMuMG revealed a significant increase in *HK2* (*p* = 0.003), *PFKFB3* (*p* = 0.0002), hexokinase 2 (*HK2*) and 6-phosphofructo-2-kinase/fructose-2,6-bisphosphatase 3 (*PFKFB3*); these changes are consistent with an increased glycolytic flux towards the production of lactate (Supplementary Table S3). In addition, we found a significantly increased expression of *HAS2*, which encodes hyaluronan synthase 2 (Supplementary Table S3). The latter enzyme catalyzes the synthesis of hyaluronic acid using intracellular glucuronic acid and N-acetylglucosamine, the products of UDP-glucuronate and UDP-GlcNAc, respectively. On the other hand, we observed downregulation of the genes involved in pyruvate transfer to mitochondria and the tricarboxylic acid (TCA) cycle (*MPC1*, *MPC2*) or its conversion to acetyl-coenzyme A (AcCoA) (*PDK1-4)* (Supplementary Table S3).

Alongside glycolysis, altered TCA cycle activity has also been suggested to be associated with EMT [28]. Here, we found that TGF-β treatment resulted in a marked increase in all the identified TCA cycle intermediates, with fumarate and malate showing the most notable increase (*p* < 0.01) (Figure 2B, Figure S2 and Supplementary Table S2) Consistent with citrate and malate increase, aspartate also exhibited a higher intracellular concentration accompanied with a significant secretion to the culture medium and increased production of asparagine. Replenishment of the TCA cycle may occur from acetyl-coenzyme A (AcCoA) produced by the β-oxidation of free fatty acids (FA). However, we observed that TGF-β resulted in significantly less intracellular acetylcarnitine (*p* < 0.001; Figure 2B), which is generated from FA catabolism. Furthermore, glutamine could also feed into the TCA cycle via the conversion to glutamate and the subsequent deamination of glutamate to α-ketoglutarate. Although we did not observe a significant difference in glutamine consumption, we found higher intracellular glutamine and glutamate (*p* < 0.01) levels in TGF-β-treated NMuMG cells. Interestingly, it has been shown that glutamate dehydrogenase 1 (GLUD1), which catalyzes the reversible conversion of glutamate to α-ketoglutarate, is reduced in NMuMG cells upon treatment with TGF-β [29].

Overall, the addition of TGF-β resulted in marked changes in the intracellular pools of metabolites participating in central carbon metabolism. Next, we performed pathway enrichment analysis to identify other significantly affected metabolic pathways. We included all metabolite concentrations and the (Kyoto Encyclopedia of Genes and Genomes) (KEGG) *Mus musculus* (mmu) metabolic pathways database [30]. The resulting pathway mapping implies that TGF-β affects the metabolism of inositol phosphate (IP); the phosphatidylinositol signaling system (PI); and the metabolism of glycine, serine, and threonine (GST), arginine and proline (RP), taurine and hypotaurine (Tau/HyTau), alanine, aspartate, and glutamate (ADE); and the glycerophospholipid metabolism (GPL) (Figure 2C; Supplementary Table S2).

### 2.3. TGF-β-Induced Depletion of Myo-Inositol and Increase in Putrescine and Proline Levels in NMuMG Cells

**Figure 3:**
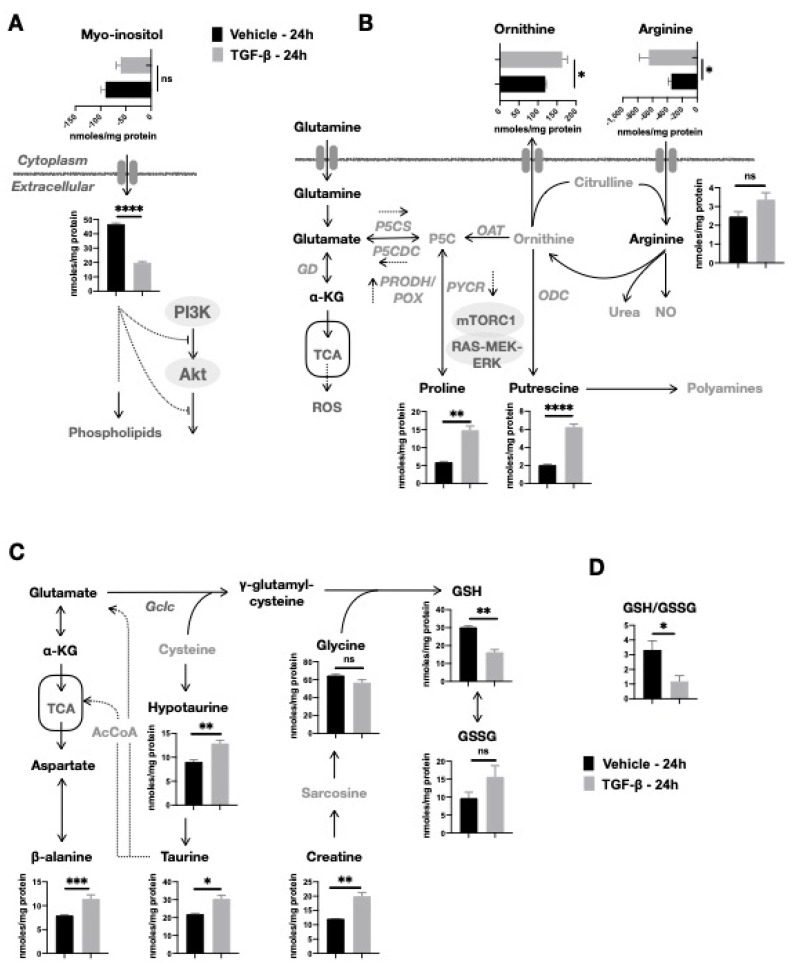
Effect of TGF-β on myo-inositol, putrescine, proline levels, and redox state. (**A**) TGF-β decreases Myo-inositol in NMuMG cells. (**B**) TGF-β (5 ng/mL) treatment induced significant changes in Arginine–Proline metabolism, resulting in synthesis of putrescine. (**C**) Glutathione disulfide (GSSG) levels were higher after TGF-β (5 ng/mL) treatment and, in contrast, there were reduced glutathione (GSH) levels. TGF-β induced hypotaurine and its product taurine level. The former is synthesized from cysteine when the latter is not used for the synthesis of glutathione, while taurine can be further directed to several pathways. (**D**) TGF-β (5 ng/mL) treatment reduced the GSH/GSSG ratio (on the *y*-axis), which was reduced in NMuMG cells. Results are shown as mean ± s.d; * *p* < 0.05; ** *p* < 0.01; *** *p* < 0.001; **** *p* < 0.0001; ns, non-significant.

We found that TGF-β induced a marked decrease in myo-inositol (*p* < 0.0001) and an increase in putrescine (*p* = 0.0003) and proline (*p* = 0.0015) in NMuMG cells. Myo-inositol is a cyclohexane polyol, which is converted within the cells into inositol phospholipids. The significantly lower intracellular myo-inositol levels upon TGF-β treatment might as well indicate its increased utilization for the synthesis of inositol phospholipids. However, we also observed less consumption (*p* = 0.0871) of myo-inositol from the culture medium despite its availability (Figure 3A). Thus, we cannot exclude the possibility that the reduced intracellular myo-inositol might occur due to TGF-β induced blockage of its import, rather than the increased demand for the synthesis of inositol phospholipids.

Furthermore, the increase in putrescine induced by TGF-β suggested an upregulation of the biosynthetic pathway of polyamines. Putrescine is the simplest polyamine in mammalian cells, and it is the product of arginine metabolism and its conversion to ornithine upon the action of Arginase 1 (ARG1) as part of the urea cycle. Ornithine in turn, is decarboxylated by ornithine decarboxylase (ODC) for the biosynthesis of putrescine (Figure 3B). Our data show an increased consumption of arginine in TGF-β-treated NMuMG cells (*p* = 0.0879) and secretion of ornithine (*p* = 0.0274), which, together with the significant increase in putrescine, suggest an upregulated ODC activity. In agreement with this, we found the expression of ODC to be significantly higher in TGF-β-treated NMuMG cells for 24 h (*p* = 0.0415) compared to vehicle NMuMG cells (Supplementary Table S3).

We also observed a marked increase in intracellular proline in TGF-β-treated NMuMG cells and no uptake or secretion to the culture medium. Proline may originate from either glutamate or from ornithine, which is produced by arginine, via pyrroline-5-carboxylate (P5C) (Figure 3B). P5C, being both a precursor and the first degradation product of proline, is a central intermediate allowing the carbon transfer between proline metabolism, the urea cycle, and the TCA cycle. Proline biosynthesis from P5C is catalyzed by pyrroline-5-carboxylate reductase (Pycr), while proline degradation to P5C utilizes proline dehydrogenase and proline oxidase (Prodh/Pox). While an increase in Pycr in NMuMG after TGF-β treatment has been reported, in agreement with our findings, the activity of Prodh/Pox has shown contradictory results [29].

### 2.4. TGF-β Stimulates an Oxidative Cellular Redox State

It is noteworthy that the interplay between proline metabolism and TCA cycle via glutamate intracellular pools, as NMuMG cells acquire the TGF-β-induced mesenchymal phenotype, might also be related to glutathione synthesis. In TGF-β-treated NMuMG cells, we found a significant decrease in reduced glutathione (GSH), while its oxidized form (GSSG) was increased (Figure 3C). Such a reduction of the GSH-to-GSSG ratio (Figure 3D) is indicative of an oxidative redox state, which could be the consequence of increased production of reactive oxygen species (ROS). However, an increased biosynthesis of glutathione upon TGF-β treatment is incompatible with the following observations. First, we observed a notable increase in hypotaurine and taurine after 24 h TGF-β activation (Figure 3C). Both metabolites are produced from cysteine once it is not being used for the biosynthesis of glutathione. Taurine can be further directed to pathways involved in anaplerosis of the TCA cycle, either via the synthesis of Acetyl-CoA or the conversion of glutamate to α-ketoglutarate. In addition, both TGF-β-treated and untreated NMuMG consumed comparable amounts of cystine (Supplementary Table S2), the precursor of intracellular cysteine, suggesting that there was not a TGF-β-induced shift towards increased production of GSH despite the availability of cysteine and glutamate. Further support to this is provided by the reduced expression of glutamate-cysteine ligase catalytic subunit (Gclc), a rate-limiting enzyme of glutathione synthesis, in NMuMG cells upon 24 h treatment with TGF-β [29,31]. Second, we also found β-alanine levels to be markedly increased, which is an indication of reduced oxidative stress [32]. Thus, while TGF-β-induced higher oxidation of GSH to GSSG, our data suggest that glutathione biosynthesis is reduced in TGF-β-treated NMuMG cells.

### 2.5. TGF-β Affects Choline Metabolism

**Figure 4:**
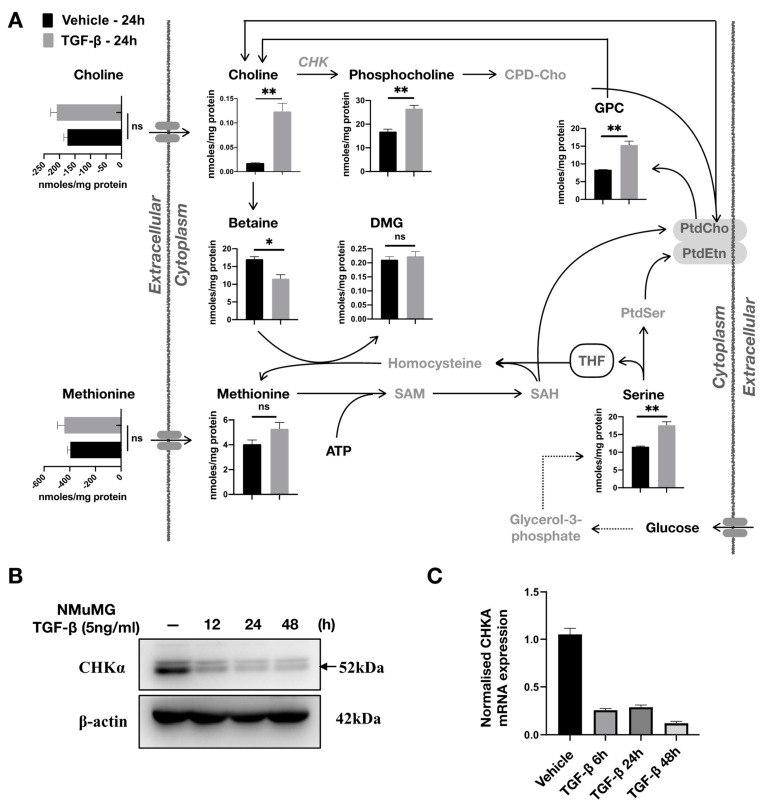
Effects of TGF-β on choline metabolism and CHKα expression. (**A**) Uptake of extracellular methionine and choline was not affected after TGF-β (5 ng/mL) treatment; however, intracellular choline and its direct product phosphocholine and indirect intermediate sn-glycerophosphocholine (GPC) were significantly increased. Betaine, also synthesized by choline, was decreased upon treatment of NMuMG cells with TGF-β (5 ng/mL). Results are shown as mean ± s.d; * *p* < 0.05; ** *p* < 0.01; ns, non-significant. (**B**) TGF-β (5 ng/mL) treatment reduced the CHKα protein expression in NMuMG cells. (**C**) TGF-β (5 ng/mL) reduced *CHKα* mRNA expression in NMuMG cells.

We found that upon treatment of NMuMG cells with TGF-β, both free intracellular choline (*p* = 0.0031) and its direct product phosphocholine (PCho) (*p* = 0.0047) were markedly increased (Figure 4A). Surprisingly, the uptake of extracellular choline was not significantly different (*p* = 0.2074) and is thus unlikely to explain the observed change in intracellular levels. On the other hand, degradation of phosphatidyl choline (PtdCho) can regenerate both PCho and free choline, the latter via the synthesis of *sn*-glycero-3-phosphocholine (GPC), which we also observed to be notably increased upon TGF-β treatment (*p* = 0.0029). Furthermore, we found betaine, which is the product of choline oxidation, to be decreased (*p* = 0.0151) hence indirectly contributing to free choline accumulation. Other possible contributions to free intracellular choline may arise from the methionine salvage pathway and the synthesis of S-adenohomocysteine (SAH) as well as from serine via the folate pathway and the production of phosphatidylserine (PtdSer) [29]. Both methionine (*p* = 0.1091) and serine (*p* = 0.0031) were found to be increased in TGF-β-treated NMuMG cells.

Based on the drastic difference in total choline (the sum of choline, PCho, and GPC) between TGF-β-treated NMuMG and control cells, we explored whether the cytidine diphosphate choline (CDP-Cho) pathway was altered by TGF-β-induced EMT. We used qRT-PCR and Western blot to measure the expression of choline kinase α (CHKα) at both mRNA and protein levels, respectively. Surprisingly, we found CHKα to be significantly decreased in mRNA and protein expression under TGF-β-induced EMT despite the increased PCho (Figure 4B,C). The reduced activity of choline phosphorylation by CHKα might also explain the significant accumulation of intracellular choline.

### 2.6. TGF-β-Induced Metabolic Reprogramming of Premalignant MCF10A-Ras Cells

**Figure 5:**
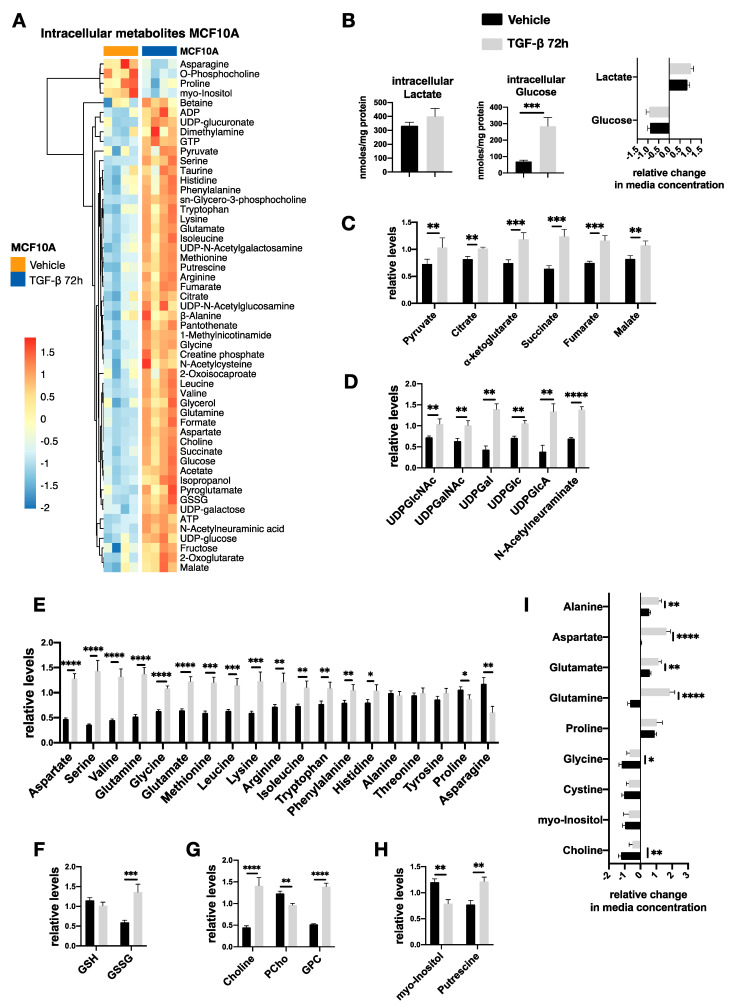
Analysis of TGF-β-induced metabolic changes in MCF10A-Ras cells after 72 h treatment. (**A**) Heatmap of standardized concentrations (z-scores) of significantly different (*p* < 0.05, n = 3) intracellular metabolites after addition of TGF-β (5 ng/mL); (**B**) While the intracellular lactate and glucose were increased with TGF-β (5 ng/mL), extracellular levels did not change. (**C**) An increase in TCA cycle intermediates were observed upon TGF-β (5 ng/mL) treatment. (**D**) Metabolites involved in hexosamine and glucuronic acid pathways were increased after TGF-β (5 ng/mL) addition. (**E**) The intracellular amino acid also increased upon TGF-β treatment. (**F**) A significant increase in glutathione disulfide (GSSG) but no differences in glutathione (GSH) were observed after TGF-β (5 ng/mL) challenge. (**G**) TGF-β (5 ng/mL) induced free choline and glycerylphosphorylcholine (GPC) accumulation, but less phosphonylcholine (PCho). (**H**) Myo-inositol and putrescine were decreased and increased in TGF-β-treated (5 ng/mL) cells, respectively. (**I**) TGF-β (5 ng/mL) induced a significant secretion of glutamine, and glutamate was observed in MCF10A-Ras cells. Results are shown as mean ± s.d; * *p* < 0.05; ** *p* < 0.01; *** *p* < 0.001; **** *p* < 0.0001; ns, non-significant.

We next tested effects on metabolic changes in human premalignant MCF10A-Ras breast cells upon treatment with TGF-β. Consistent with our findings in mouse normal NMuMG cells, we observed morphological changes and increased expression of N-cadherin and Vimentin after TGF-β stimulation [33,34] (Figure S1). Compared with NMuMG cells, the TGF-β-induced EMT in MCF10A-Ras cells occurs later (apparent after 72 h of stimulation) and less pronounced. Furthermore, TGF-β-induced metabolic reprogramming to MCF10A-Ras, which was evident at 72 h post-TGF-β administration over a time course of 96 h (Figure S3). Looking specifically at 72 h for the effects of TGF-β treatment, we found that 54 intracellular metabolites were significantly altered compared to vehicle-treated MCF10A-Ras cells (Figure 5A). While intracellular glucose and lactate levels were increased with TGF-β, consumption and secretion of glucose and lactate, respectively, were not significantly different compared to vehicle control-treated cells (Figure 5B). Furthermore, TGF-β also induced an increase in all TCA cycle intermediates (Figure 5C), as we observed in NMuMG cells. In contrast to NMuMG cells, all the products of the hexosamine and glucuronic acid pathway were increased with TGF-β (Figure 5D), and we also observed increased intracellular pools of most amino acids with a notable exception of proline in MCF10A-Ras cells (Figure 5E). Proline synthesis was found to be induced by TGF-β in NMuMG cells; however, in MCF10A-Ras, intracellular proline was lower compared to vehicle, despite higher proline uptake from the culture medium (Figure 5I). Another notable difference of MCF10A-Ras was significant secretion of glutamine and glutamate in TGF-β-treated cells (Figure 5I), while we also found a significant increase in GSSG but no difference in GSH (Figure 5F). Although these findings point to different TGF-β-induced metabolic adaptation between the NMuMG and MCF10A-Ras cells, we did find similar results with regard to choline metabolism, myo-inositol, and putrescine. Specifically, TGFβ induced free choline and GPC accumulation in MCF10A-Ras cells, but decreased PCho (Figure 5G), while there was no difference in choline uptake (Figure 5I). In addition, upon TGF-β treatment the myo-inositol and putrescine levels were significantly decreased and increased, respectively (Figure 5H). Taken together, these results suggested that some metabolic pathways such as polyamine synthesis and choline metabolism are affected by TGF-β in both NMuMG and MCF10A-Ras cells during EMT.

### 2.7. TGF-β-Induced Phosphocholine Production may Play a Role in EMT

**Figure 6:**
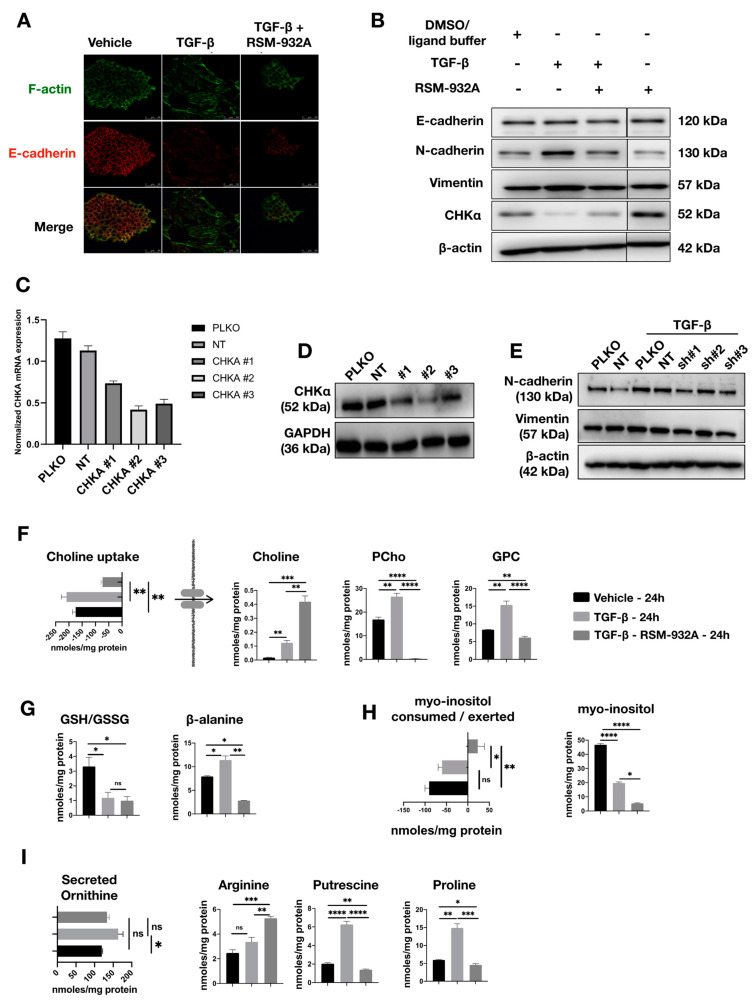
Effect of TGF-β in the absence or presence of RSM-932A on TGF-β-induced EMT, phosphocholine, and myoinositol metabolism. (**A**) Treatment with RSM-932A (10 μM) inhibited the F-actin stress fiber formation and re-localization of E-cadherin that was induced by TGF-β (5 ng/mL) treatment with 24 h. (**B**) The increased expression of N-cadherin and Vimentin induced by TGF-β (5 ng/mL) were downregulated with RSM-932A (10 μM) treatment. (All samples were run on the same gel). (**C**) The knockdown efficiency of *CHKA* in mRNA level in NMuMG cell line. (**D**) The knockdown efficiency of CHKα in protein levels in NMuMG cells. (**E**) *CHKA* knockdown decreased TGF-β-induced N-cadherin and Vimentin expression. (**F**) Addition of the CHKα inhibitor RSM-932A (10 μM) together with TGF-β (5 ng/mL) completely halted the production of O-phosphocholine by choline. Furthermore, both choline uptake from the media and choline’s intracellular levels were reduced and increased, respectively. (**G**) Inhibition of CHKα did not induce any effect in the glutathione (GSH)/glutathione disulfide (GSSG) ratio (ratio in nmoles/mg protein on *y*-axis), while a marked decrease in β-alanine was observed. (**H**) Myo-inositol was decreased with TGF-β (5 ng/mL) and RSM-932A (10 μM) co-treatment, and a secretion of myo-inositol to the culture medium was observed. (**I**) Putrescine and proline were decreased upon TGF-β (5 ng/mL) and RSM-932A (10 μM) co-treatment, while arginine was notably increased. Results are shown as mean ± s.d; * *p* < 0.05; ** *p* < 0.01; *** *p* < 0.001; **** *p* < 0.0001; ns, non-significant.

Membrane reorganization is a major part of EMT, and the choline pathway is essential to generate various phosphatidylcholine-containing membrane lipids. Therefore, we evaluated the role of CHK⍺ activity in TGF-β-induced EMT by inhibiting CHKα with RSM-932A. RSM-932A is the first CHKα inhibitor to be tested in patients with advanced solid tumors in a phase I clinical trial conducted by TCD Pharma (Valladolid, Spain) at two U.S. clinical centers (NCT01215864). We found that RSM-932A inhibited TGF-β-induced F-actin stress fiber formation of NMuMG cells, as well as the TGF-β-induced re-localization of E-cadherin (Figure 6A). We also observed a reduced TGF-β-induced expression of mesenchymal markers N-cadherin and Vimentin in response to RSM-932A treatment in NMuMG and MCF10A-Ras cells (Figure 6B and Figure S4). Surprisingly, TGF-β decreased the CHKα levels, and this effect was partly inhibited by RSM-932A treatment (Figure 6B). To further confirm whether selective inhibition of CHKα function can inhibit TGF-β-induced EMT, *CHKA* was targeted with three different shRNAs. This resulted in decreased *CHKA* mRNA and protein expression (Figure 6C,D). Whereas the genetic *CHKA* knockdown slightly decreased TGF-β-induced N-cadherin expression, the TGFβ-induced morphological change and F-actin stress fiber formation were not affected (Figure 6E). This might be attributed to imperfect genetic knockdown efficiency and/or a requirement of CHKα in only a part of the EMT-linked responses.

Since the addition of RSM-932A impairs TGF-β-induced EMT, we asked whether it also affected additional TGF-β-mediated metabolic changes. As expected, we found PCho to be depleted while free intracellular choline was accumulated since it was not utilized and its uptake was also reduced (Figure 6F). RSM232A did not induce an effect on GSH/GSSG ratio; however, the marked decrease in β-alanine might indicate increased oxygen consumption (Figure 6G). Interestingly, myo-inositol levels were further decreased with TGF-β and RSM-932A co-treatment, while we also observed a secretion of myo-inositol to the culture medium (Figure 6H). Notably, we observed a marked decrease in putrescine and proline in TGF-β and RSM-932A co-treatment, almost to the levels of untreated NMuMG cells, while arginine was notably increased (Figure 6I). Since proline and putrescine biosynthesis are not directly related to CHKα activity, we examined whether their intracellular levels were also affected by RSM-232A in the absence of TGF-β stimulation. We found 43 metabolites to be significantly different (Table S3) in RSM-232A-treated NMuMG, suggesting that the inhibitor affects NMuMG metabolism in more pathways than just CHKα inhibition. Among them, we found proline to be decreased (*p* = 0.0107) compared to vehicle levels, while putrescine levels were not significantly changed (*p* = 0.2380). Collectively, our data showed that treating TGF-β-challenged NMuMG cells with RSM-932A (which inhibits the conversion of choline phosphorylation to PCho among other metabolic changes) resulted in a mitigation of some of the TGF-β-induced EMT related changes.

## 3. Discussion

Numerous studies have indicated that EMT is linked with cellular metabolic rewiring, which supports the increased energy demands of motility and invasiveness [16,17]. In this study, we generated a quantitative metabolic map of TGF-β-mediated metabolic rewiring in normal NMuMG and premalignant MCF10A-Ras breast epithelial cells, both of which underwent EMT upon TGF-β stimulation. Among the several changes in intracellular and extracellular metabolite pools, the most pronounced ones were related to polyamine and proline biosynthetic pathways and the choline cycle activity. These three metabolic pathways, as expressed by the biosynthesis of putrescine and proline and the total choline pool, were highly affected by TGF-β stimulation.

Very recently, it was shown that increased activity of the cytidine diphosphate ethanolamine (CDP-Etn) pathway, which produces phosphatidyl ethanolamine (PE) from free ethanolamine, was correlated with increased mesenchymal-to-epithelial transition (MET) in induced pluripotent stem cells (iPSCs) [35]. It was also shown that inhibition of the CDP-Etn pathway via either ethanolamine (Etn) or phosphatidyl-ethanolamine (Ptd-Etn) resulted in an increased expression of mesenchymal markers, thus reversing MET to EMT. Here we observed an increase in the concentration of total intracellular choline, i.e., the sum of free intracellular choline, PCho, and GPC, when we induced EMT in both the mouse NMuMG epithelial and the human premalignant MCF10A-Ras breast cells after 48 h and 96 h, respectively. PCho is both a precursor and a product of Ptd-Cho; PCho and Ptd-Etn are the major lipid components of cell membranes and are produced via two mirror pathways, CPD-Cho and CPD-Etn. CHKα, which phosphorylates choline to PCho, is a key regulator of CPD-Cho activity and is related to tumor metastasis and drug resistance [36,37]. CHK⍺ inhibition suppressed EMT in glioblastoma and breast cancer cells [38,39]. In our study, while TGFβ-induced EMT significantly downregulated protein and mRNA expression of CHKα, pharmacological inhibition of CHKA activity increased the expression of CHKα and decreased the expression of mesenchymal marker N-cadherin. It was recently suggested that the non-catalytic expression of CHKα is important in promoting cancer cell survival and that this expression is independent of its catalytic activity [40]. Thus, it is possible that the increased expression of CHKα as triggered by the inhibitor RSM-932A induces a negative feedback regulation in TGF-β-triggered EMT, accompanied by decreased choline phosphorylation to produce PCho (Figure 6F). Additionally, it is possible that CHKα plays a scaffolding function role in inhibiting EMT induced by TGF-β. Moreover, it has been demonstrated that inhibition of CHKA can reduce the motility and invasive ability of cancer cells [38,41]. In line with this, highly metastatic MDA-MB-231 breast cancer cell line showed impaired migration independent of proliferation and decreased in vivo invasion in zebrafish xenograft assays when *CHKA* was knocked down (Figure S5A–F).

In another recent study, it was demonstrated that TGF-β orchestrates fibrogenic and developmental EMT via the RAS effector RREB1 [42]. RREB1 activation by mitogen-activated protein kinase (MAPK) recruits TGF-β-activated SMAD factors to SNAIL, a transcriptional factor that drives EMT, which provides a molecular link between RAS and TGF-β pathways for coordinated induction of EMT. Furthermore, it was shown previously that polyamine metabolism is controlled by the RAS-MEK-ERK signaling pathway, AKT signaling, and the PTEN-PI3K-mTOR complex 1 (mTORC1) [43]. Moreover, inhibition of mTORC1 in both mouse and human prostate tumors has markedly reduced polyamine biosynthesis [44]. In relation to our findings, putrescine biosynthesis from both arginine and ornithine, as well as proline biosynthesis, was promoted by TGF-β and was severely affected by inhibition of EMT (Figure 6I). The concentrations of both metabolites were returned to the range of values seen in untreated NMuMG cells. Further evidence regarding the critical role of proline in EMT [45] came from the recent finding that proline metabolism can support metastasis of cancer cells, as studied in MCF10A H-Ras cells [46]; additionally, increased activity of PI3K markedly upregulates enzymes controlling proline biosynthesis [47]. Thus, targeting proline metabolism could provide promising avenues to interfere with TGFβ-induced EMT.

In addition to the metabolic changes described above, we also observed a metabolic rewiring involving glycolysis and the TCA cycle. Glycolysis has a key role in EMT by providing the necessary building blocks and energy for macromolecular anabolism. Enhanced glycolysis promoted cell motility and high levels of lactate were found to be directly related to metastasis in several cancers [48]. It has been shown that TGF-β increases glycolytic rates [18,19] and, accordingly, we have shown TGF-β to promote glycolysis during EMT in NMuMG cells, and to a lesser degree in MCF10A-Ras cells, resulting in the accumulation of lactate levels, which in turn contributes to the EMT process. Furthermore, dysregulation of TCA cycle has often been described to occur during EMT, especially via alterations in the activity of fumarate hydratase (FH), succinate dehydrogenase (SDH), and isocitrate dehydrogenase (IDH) [22,24,25]. We have found a marked increase in TCA cycle intermediates, especially fumarate and malate. Interestingly, inhibition of EMT by RSM-932A resulted in a marked decrease in citrate, α-ketoglutarate, succinate and fumarate, and intracellular glutamate and aspartate, but not malate (Figure S6). However, we should be cautious when drawing conclusions regarding EMT inhibition by RSM-932A as this inhibitor may also target other metabolic pathways in addition to choline phosphorylation, as demonstrated by the numerous RSM-932A-induced metabolic changes in NMuMG cells (Figure S7, Table S3). However, with regard to TCA cycle activity, we did not evaluate the effect of TGFβ-induced EMT on the enzymes involved (e.g., FH, SDH and IDH), thus it is possible that the accumulated intracellular pools of TCA cycle intermediates is the result of TCA cycle dysfunction rather than an increased activity, which is triggered by TGF-β or progressively occurring during TGF-β induced EMT [28]. Further work is needed in this area, especially utilizing metabolic flux analysis models to reveal the TCA cycle dynamics and the fluxes of intermediates through the intracellular compartments. Nonetheless, this study demonstrates that our quantitative metabolic map of TGF-β-induced metabolic rewiring provides a basis for future studies on the involvement of changes in metabolites in TGF-β-induced responses.

## 4. Materials and Methods

### 4.1. Cell Culture

Mouse immortalized NMuMG breast epithelial cell line [31], human MDA-MB-231 breast cancer cell line, and human 293T embryonic kidney (HEK) cells were purchased from ATCC. These cell lines were cultured in Dulbecco’s modified Eagle medium (DMEM, 11965092, Thermo Fisher Scientific, Waltham, MA, USA) with 10% fetal bovine serum (FBS, 16000044, Thermo Fisher Scientific). MCF10A-derived cell line MCF10A-Ras (M2) was kindly provided by Dr. Fred Miller (Barbara Ann Karmanos Cancer Institute, Detroit, MI, USA) [26,36], cultured in DMEM/F12 medium (11039047, Thermo Fisher Scientific) supplemented with 5% horse serum (26050088, Thermo Fisher Scientific), 20 ng/mL epidermal growth factor (EGF, 01-107, Merck Millipore, Burlington, VT, USA), 10 mg/mL insulin (91077C, Sigma-Aldrich, St. Louis, USA), 100 ng/mL cholera enterotoxin (C8052, Sigma-Aldrich), 0.5 mg/mL hydrocortisone (H0135, Sigma-Aldrich). In addition to the TGF-β-induced EMT response in both cell lines, TGF-β also induces cytostatic effects in NMuMG and MCF10A-Ras cells (albeit stronger in NMuMG than in MCF10-A -RAS cells). All cell lines were frequently tested for the absence of mycoplasma contamination. Authenticity checks on human cell lines were performed using the short tandem repeats (STR) profiling method. Recombinant human TGF-β3 was a kind gift of Andrew P. Hinck, University of Pittsburg, USA. RSM-932A was purchased from Cayman (Item No. 21518).

### 4.2. Sampling NMuMG and MCF10A-Ras Cells for NMR Analysis

In short, 1.5 × 10^6^ NMuMG cells (cultured in DMEM with 10% FBS) were seeded in quadruple in a 10 cm petri dish and were grown to 50–60% confluency before treatment with TGF-β (5 ng/mL) for 12, 24, or 48 h. Additionally, 1.5 × 10^6^ NMuMG cells (n = 3) were also prepared and treated with TGF-β (5 ng/mL) or RSM-932A (10 μM) for 24 h. A total of 0.1 × 10^6^ MCF10A-Ras cells (cultured in DMEM/F12 with 5% horse serum) were seeded in quadruple in 10 cm petri dish, treated with TGF-β (5 ng/mL) for 72 h after grown to 30–40% confluency.

### 4.3. NMR-Based Metabolomics

Samples for NMR analysis were prepared as previously described [26]. For measurements of extracellular metabolites, 0.3 mL of culture medium was collected from each sample prior to harvesting and mixed immediately with 0.9 mL 100% liquid chromatography (LC)-grade pre-chilled (−80 °C) methanol (MeOH). Samples were then vortexed, stored at −80 °C for at least 30 min to allow for precipitation of macromolecules, and subsequently centrifuged at 16 × 10^3^× *g* at −4 °C for 20 min. The supernatant containing the extracellular metabolites was collected and dried under gentle nitrogen stream and stored at −80 °C. For the analysis of the intracellular metabolites, the remaining growth medium in each sample was quickly removed, and cells were washed once with cold (4 °C) phosphate-buffered saline (PBS) and snap-frozen with ~10 mL liquid nitrogen. The whole process of washing and quenching took less than 10 sec per sample. Intracellular metabolites were then extracted with 1.5 mL cold solution of methanol/chloroform/water, 6.75:0.75:2.5 (*v*/*v*/*v*). Cells were lysed using a cell scraper, and both extracts and cell debris were collected into 2 mL centrifuge tubes and placed on dry ice for at least 15 min, followed by centrifugation at 16 × 10^3^× *g* at −4 °C for 10 min. The supernatants containing the intracellular metabolites were dried under gentle nitrogen stream and stored at −80 °C until further analysis. All remaining pellets after centrifugation were dissolved in an extraction solution of 8 M urea and 1 M Tris-HCl, 1:1 (*v*/*v*) (pH 8.0), and 1% SDS, and total protein concentration was determined using a DC^TM^ Protein Assay Kit (5000111, Bio-Rad).

For NMR measurements, all dried extracts were reconstituted in 250 μL of 0.15 M K_2_HPO_4_/KH_2_PO_4_ buffer (pH 7.4) in 99.9% deuterated water (D_2_O), including 0.2 mM NaN_3_ and 0.05 mM or 0.2 mM trimethylsilylpropionic-*d*_4_ acid sodium salt (TSP-*d*_4_) for cell extracts or culture medium extracts, respectively. Of each sample, 190 μL was transferred to 3 mm NMR tube for quantitative NMR analysis. The remaining 60 μL from each sample was pooled and used for 2D NMR experiments.

NMR data were recorded on a 14.1 T NMR spectrometer (600 MHz for ^1^H; Bruker Avance II) under standardized conditions for all samples. Per sample, a 1D ^1^H spectrum was collected using the *noesygppr1d* pulse sequence (TopSpin 3.0) with a mixing time of 0.01 ms, a relaxation delay of 4 sec, acquisition time of 2.65 s, and a spectral width of 12 kHz (20 ppm for ^1^H). The spectral data were phase and baseline corrected and referenced to TSP-*d*_4_ methyl protons at *δ* 0.00 ppm and subsequently imported in Chenomx NMR suit 8 (Chenomx Edmonton, AB, Canada) for quantification of metabolites. Assignment of metabolites was based on the Bruker Bbiorefcode and Chenomx databases. The structures of all annotated metabolites were then confirmed in the 2D NMR experiments of the pooled samples. The quantification of each metabolite was performed by integration of its proton peaks in the NMR spectrum using the deconvolution fitting algorithm of Chenomx NMR Suit. The calculated integrals were subsequently transformed to concentrations (mM) based on the known concentration of the internal standard TSP-*d*_4_ in each sample (0.05 mM for cell extracts and 0.2 mM for culture medium extracts). Concentrations of intracellular metabolites were transformed to nmoles based on the sample volume and normalized to the total protein mass of each sample. For extracellular metabolites, nmoles of metabolites consumed or excreted by the cells were first calculated based on the quantified concentrations in the NMR samples using the formula (C*_spent_* − C*_fresh_*) × V_NMR_ × 0.001, where C*_spent_* (mM) is the concentration of each metabolite as quantified in the culture medium after incubation, C*_fresh_* (mM) is the concentration of the same metabolite in parallel culture of triplicate cells-free media, and V_NMR_ (mL) is the volume of the NMR sample. Subsequently, the extracted nmoles were corrected to the volume of each cell culture (7 mL) and normalized to the total protein weight (mg) of each sample. A positive quantity of nmoles metabolite per mg of protein indicates excretion, while a negative value indicates consumption.

### 4.4. Lentiviral Transduction

Lentiviruses were produced by co-transfecting shRNAs with the helper plasmids pCMV-VSVG (vesicular stomatitis virus glycoprotein), pMDLg-RRE (gag/pol), and pRSV-REV (regulator of virion expression) into HEK293T cells using polyethyleneimine (PEI). Cell supernatants were harvested 48 h after transfection and stored at −80 °C. Puromycin (2 μg/mL, P9620, Sigma-Aldrich) was used to maintain selection pressure. Mouse and human CHKA lentiviral shRNAs were obtained from the Sigma MISSION shRNA library. Generally, five shRNAs were tested, and two or three most effective were used in experiments. TRCN0000024604 (sh#1), TRCN0000024606 (sh#2), and TRCN0000024607 (sh#3) were used for knockdown of *CHKα* in mouse NMuMG cells; TRCN0000006048 (sh#1) and TRCN0000271284 (sh#2) were used for knockdown of *CHKα* in human MDA-MB-231. As controls, an empty PLKO vector or non-targeting vector was used.

### 4.5. RNA Isolation and Quantitative Real-Time PCR (qRT-PCR)

Total RNAs were prepared using the NucleoSpin RNA II kit (740955, BIOKE’) according to manufacturer’s instructions. cDNA was synthesized using 1 μg RNA according to the manufacturer’s instructions of the RevertAid First Strand cDNA Synthesis Kit (K1621, Thermo Fisher Scientific). Real-time (RT) polymerase chain reaction (PCR) was performed with GoTaq qPCR Master Mix (A6001, Promega, Leiden, The Netherlands) using CFX Connect Detection System (1855201, Bio-Rad, Veenendaal, The Netherlands). All the values for target gene expression were normalized to hypoxanthine guanine phosphoribosyltransferase (*HPRT*) or glyceraldehyde-3-phosphate dehydrogenase (*GAPDH*). The sequences of primers can be found in Supplementary Table S1 in the Supplementary Data.

### 4.6. Western Blot Analysis

Cells were lysed with EBSE buffer (8% glycerol, 3%SDS, 0.1 M Tris-HCl, pH 6.8). Protein concentration was determined using a DC^TM^ Protein Assay Kit (5000111, Bio-Rad). Samples were separated by sodium dodecyl sulfate polyacrylamide gel electrophoresis (SDS-PAGE) and blotted onto a 45 μm polyvinylidene difluoride (PVDF) membrane (IPVH00010, Merck Millipore, Burlington, CA, USA); the chemiluminescent signal was detected using ClarityTM Western ECL Substrate (1705060, Bio-Rad) and ChemiDoc Imaging System (17001402, Bio-Rad). The membranes were incubated with primary antibodies against CHKα (ab88053, Abcam), N-cadherin (8220682, BD biosciences), E-cadherin (610181, BD biosciences, Allschwil, Switserland), Vimentin (5741S, Cell signaling, Leiden, The Netherlands), SLUG (9585S, Cell signaling), Tubulin (2148S, Cell signaling), GAPDH (MAB374, Merck Millipore), and β-actin (A5441, Sigma-Aldrich). Tubulin, β-actin, and GAPDH were used as protein loading controls.

### 4.7. Immunofluorescence Staining

NMuMG cells were fixed in 4% paraformaldehyde solution, permeabilized with 0.1% Triton X-100 (108643, Merck Millipore), and blocked with 3% bovine serum albumin (BSA, A2058, Sigma-Aldrich) in PBS for 1 h at room temperature (RT). The cells were incubated with primary antibodies against E-cadherin (610181, BD biosciences) in 1% BSA for 1 h at room temperature (RT). Subsequently, Alexa Fluor 488 Phalloidin (A12349, Thermo Fisher Scientific) and goat anti-mouse Alexa Fluor 555 secondary antibody (A21422, Invitrogen) were incubated together for 1 h at RT. The nuclei were stained with 4′, 6-diamidino-2-fenylindool (DAPI, 62248, Thermo Fisher Scientific). Fluorescence images were acquired using confocal microscopy (SP5, Leica Microsystems, Amsterdam, The Netherlands).

### 4.8. Cell Migration and Proliferation Assay

For migration, cells were seeded in triplicate on 96-well Essen Image Lock plates (4379, Essen BioScience, Ann Arbor, MI, USA) at 80 × 10^3^ per well with 100 μL medium and were allowed to adhere overnight. After wounding with the wound maker (Essen BioScience, Ann Arbor, USA), the medium was aspirated, and each well was washed two times with PBS followed by addition of 100 μL of medium. Analysis of wound closure was performed using the Incucyte Scratch Wound Analysis module. Images were obtained every 2 h. For proliferation, cells were also plated in triplicate on 96-well plate (3595, Essen BioScience) at 1 × 10^3^ per well with 100 μL of medium and were allowed to adhere overnight. The Incucyte Standard module was applied for proliferation analysis, and images were acquired every 2 h.

### 4.9. Statistical Analysis

Data were analyzed using GraphPad Prism 8.0 (GraphPad Software, La Jolla, CA, USA). Numerical data from triplicates are presented as the mean ± standard deviation (s.d.). Experiments were analyzed with an unpaired Student’s *t* test. *p* < 0.05 was considered statistically significant. Analysis and visualization of metabolomics data was performed in R statistical software (http://www.R-project.org/, version 4.0.3, accessed on 15 July 2021) using the packages *pheatmap, dplyr, ggplot, ggsci,* and *RColorbrewer*, as well as GraphPad Prism 8.0, San Diego, CA, USA). Between the group comparisons of metabolites, concentrations were performed using an unpaired Student’s *t* test, and *p*-values were corrected using the False Discovery Rate (FDR) approach of Benjamini, Krieger, and Yekutiely (GraphPad Prism 8, San Diego, CA, USA) with a cutoff of 5% for Q for a comparison to be considered significant. Cluster analysis of metabolites was performed in R using the *factoextra* package and the Spearman correlations.

## 5. Conclusions

Overall, our results show that TGF-β triggered metabolic reprogramming in normal NMuMG cells and premalignant MCF10A-Ras cells during EMT, mainly affecting the CDP-choline pathway and polyamine and proline biosynthesis. The treatment of cells with a kinase inhibitor RSM-93A that inhibits CHKα (among other kinases) inhibited TGF-β-induced features associated with mesenchymal phenotype and, in addition to choline metabolism, was also significantly reflected in proline and putrescine pools. Our work provides further evidence that TGF-β signaling and metabolism are intertwined.

## Data Availability

All data is available within article and supplementary material.

## Supplementary Materials

The following are available online at https://www.mdpi.com/article/10.3390/metabo11090626/s1, Figure S1: TGF-β induces EMT of MCF10A-Ras cells, Figure S2: TGF-β induced metabolic kinetic changes in intracellular metabolites in NMuMG cells, Figure S3: TGF-β induced metabolic kinetic changes in intracellular metabolites in MCF10A-Ras cells, Figure S4: Effect of RSM-932A on TGF-β induced an increase of expression of N-cadherin and Vimentin. RSM-932A was used at 10 µM and TGF-β at 5 ng/mL (All samples were run on the same gel), Figure S5: Depletion of CHKα in MDA-MB-231 cancer cells inhibited migration and extravasation, Figure S6: Effect of TGF-β in the absence and presence of RSM-932A on TCA cycle intermediates in MCF10A-Ras cells, Figure S7: Analysis of RSM-932A and/or TGF-β treatment in NMuMG cells on choline metabolism, Table S1: List of RT-qPCR primers, Table S2: Changes in extracellular metabolites in the absence or presence of TGF-β or RSM-932A, and TGF-β-induced in intracellular metabolites in MCF10A-Ras cells, Table S3: TGF-β-induced kinetic effects on gene expression encoding metabolic enzymes in NMuMG cells.
