# Supplementary material for: Metabolic Reprogramming of Mammary Epithelial Cells during TGF-β-Induced Epithelial-to-Mesenchymal Transition
Source: Metabolites. 2021 Sep 15;11(9):626. doi: 10.3390/metabo11090626 (PMC8464788; doi:10.3390/metabo11090626)
Supplement: Supplementary file 1 [file metabolites-11-00626-s001.zip › Supplementary Material Metabolic reprogramming of mammary epithelial cells during TGF rev-wan ptd.pdf]

Supplementary Material Metabolic reprogramming of mammary epithelial cells during TGF-β-induced epithelial-to-mesenchymal transition

Wan Hua[1,2*], Sarantos Kostidis[3*], Oleg Mayboroda[3], Martin Giera[3], Marten Hornsveld[1] and Peter ten Dijke[1]

Supplementary Figures

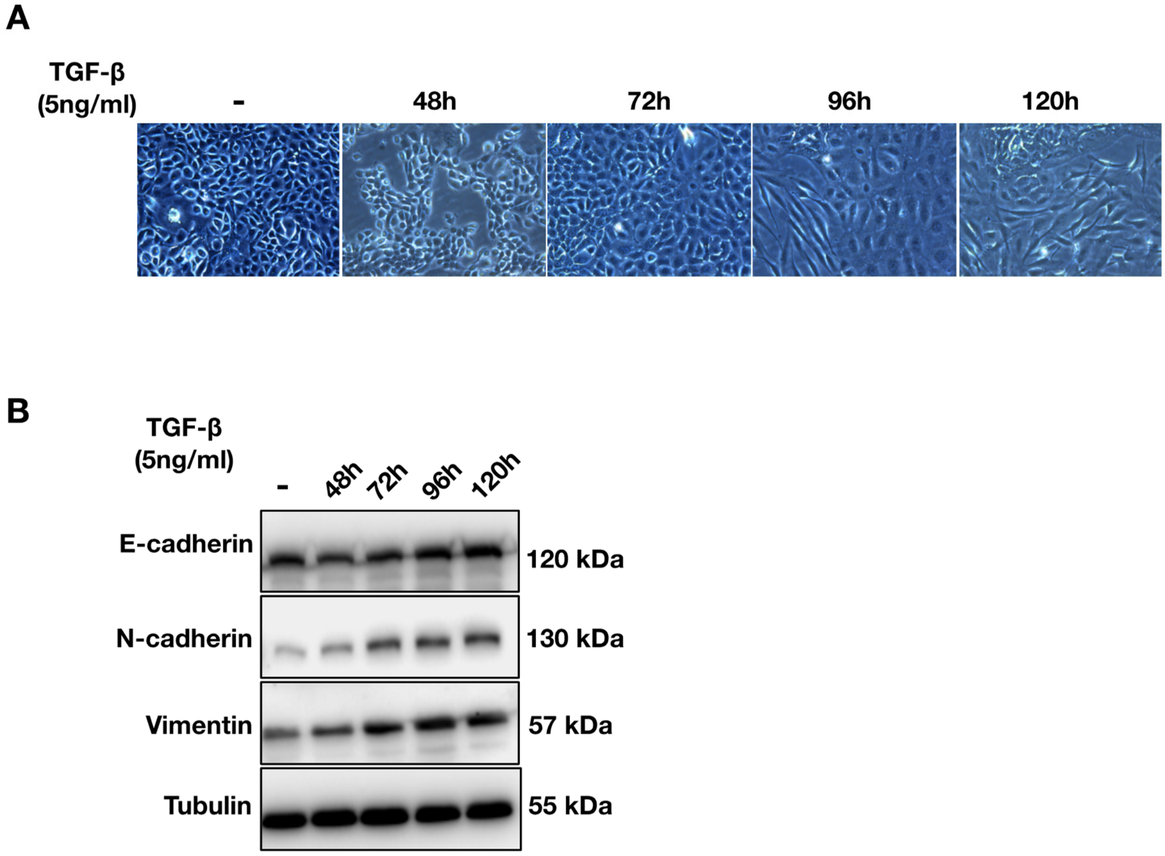

Figure S1. TGF-β induces EMT of MCF10A-Ras cells. A. TGF-β induced a morphological change in MCF10A-Ras cells from an epithelial to a spindle-shaped fibroblast-like mesenchymal phenotype. B. TGF-β induced an increase in expression of N-cadherin and Vimentin in MCF10A-Ras cells for 48 h, 72 h, 96 h and 120 h.

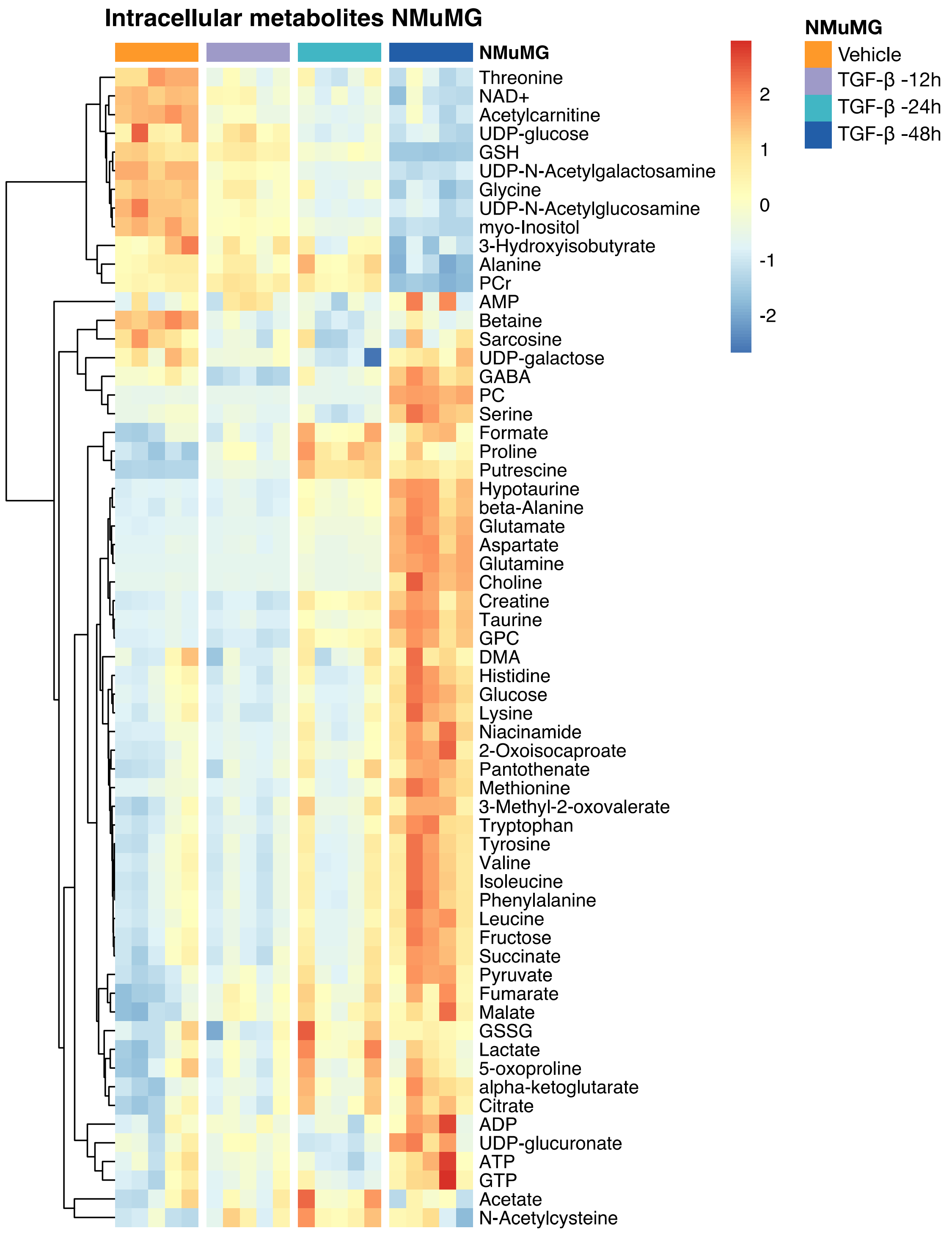

Figure S2. TGF-β induced metabolic kinetic changes in intracellular metabolites in NMuMG cells. The treatment-induced changes in intracellular metabolites are compared to vehicle control-treated cells. Results from n = 5 independent experiments are shown.

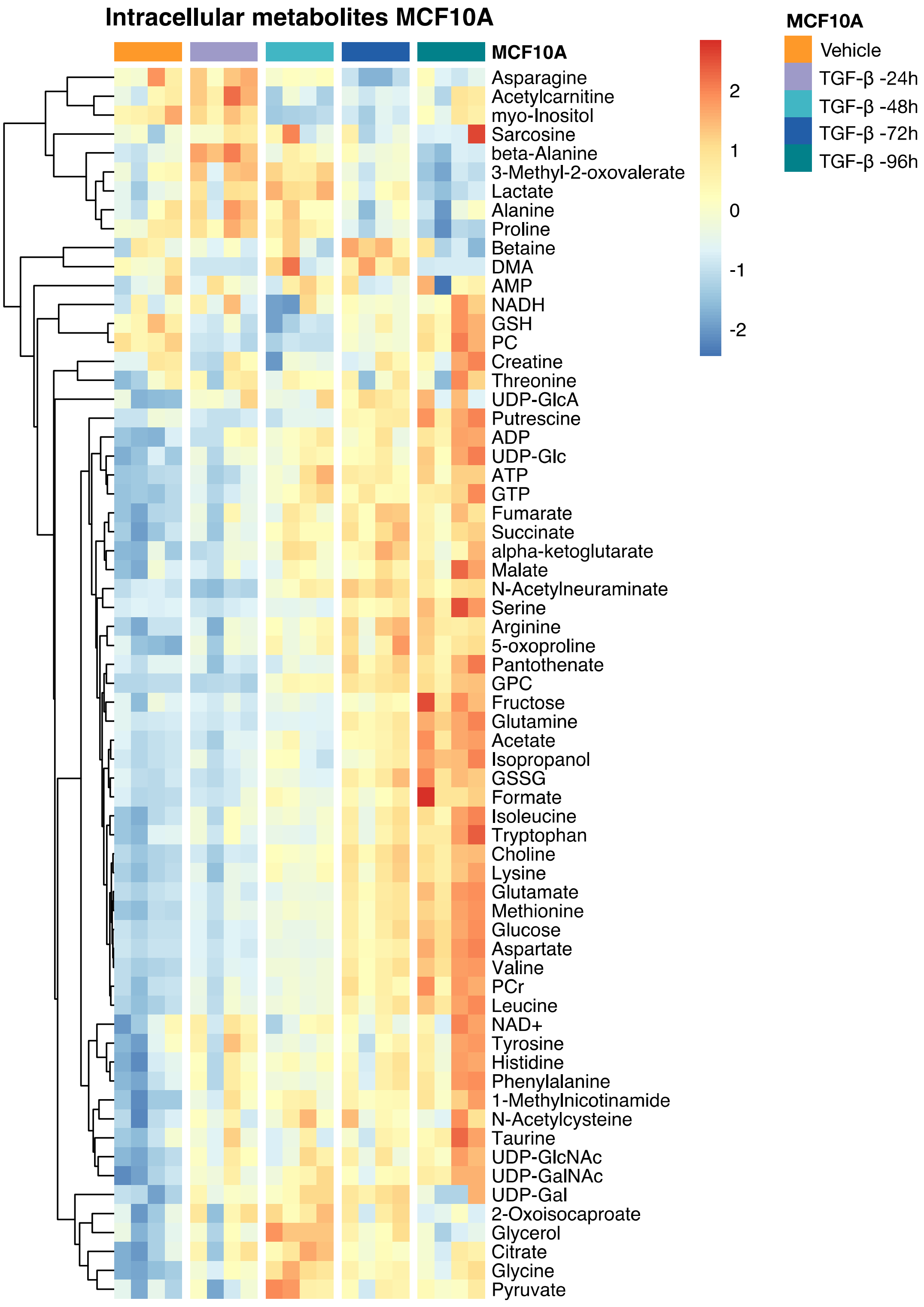

Figure S3. TGF-β induced metabolic kinetic changes in intracellular metabolites in MCF10A-Ras cells.

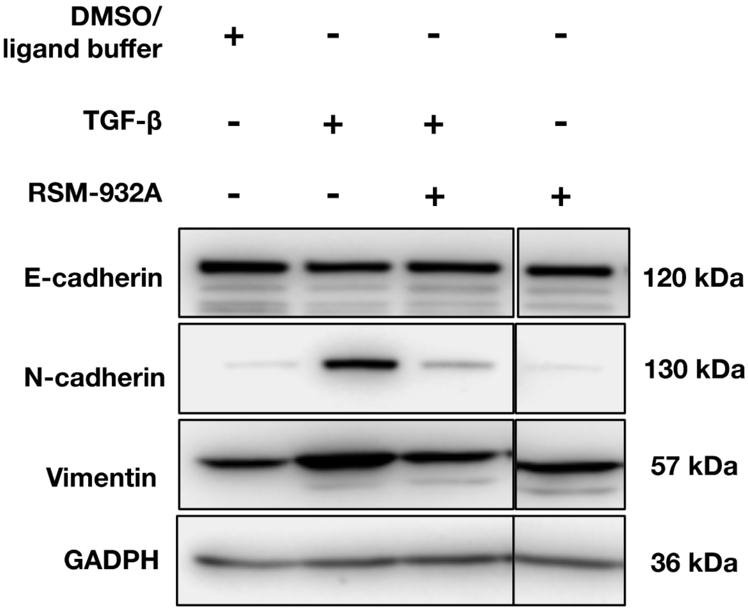

Figure S4. Effect of RSM-932A on TGF-β induced an increase of expression of N-cadherin and Vimentin. RSM-932A was used at 10μM and TGF-β at 5ng/mL (All samples were run on the same gel).

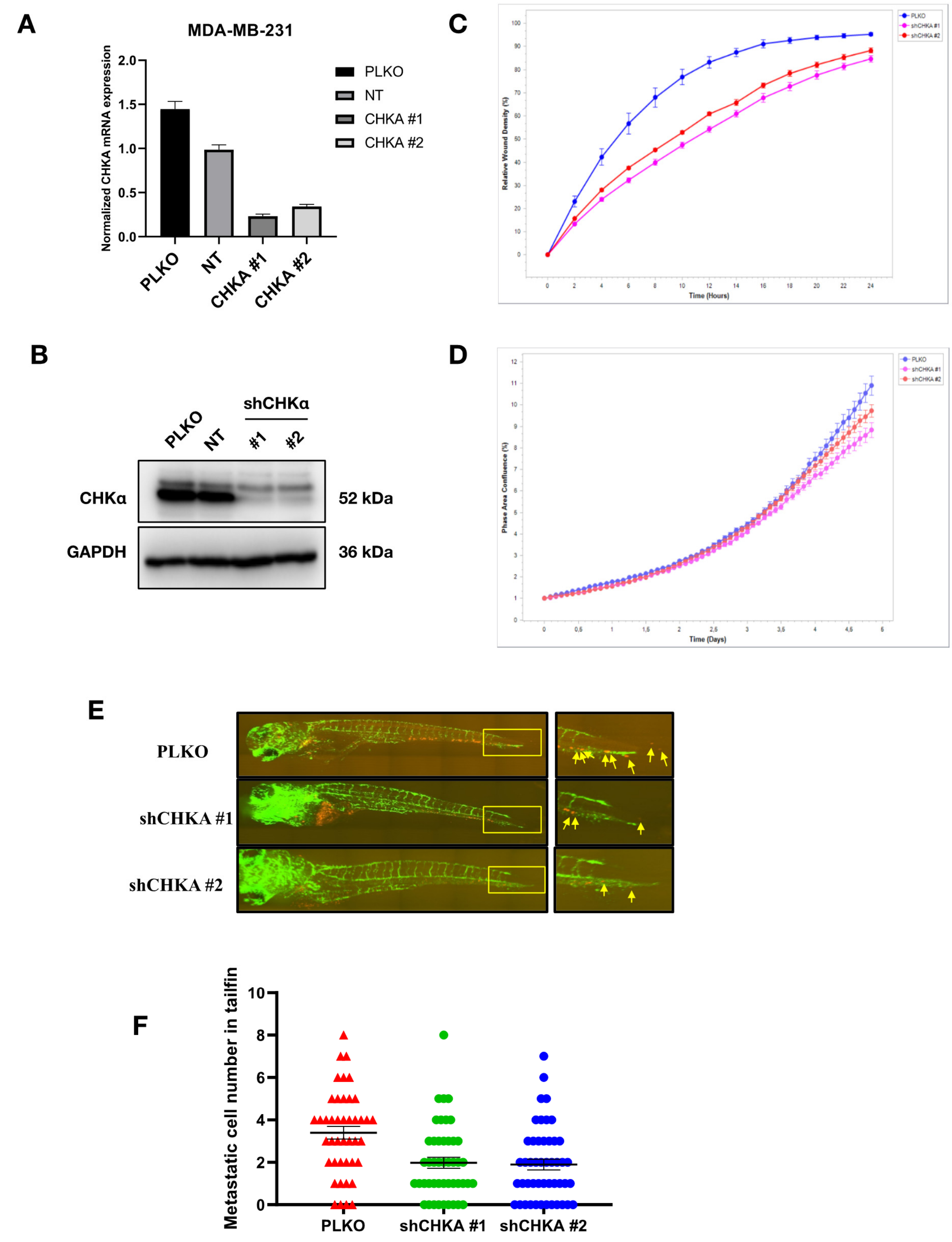

Figure S5. Depletion of CHKα in MDA-MB-231 cancer cells inhibited migration and extravasation. A. shRNA targeting *CHKα* inhibit its mRNA expression. PLKO, empty vector control; NT, non-targeting control. B. shRNA targeting *CHKα* inhibit its encoded protein expression. PLKO, empty vector control; NT, non-targeting control. C. Effect of shRNA-mediated *CHKα* knockdown on MDA-MB-231 cell proliferation was not significantly

affected. The proliferation was measured by analyzing the cell confluency using Incucyte standard module. PLKO-infected cells were used as control. D. Inhibitory effect of shRNA-mediated *CHKα* depletion on MDE-MB-231 cell migration. Migration was measured using Incucyte scratch wound analysis module. PLKO-infected cells were used as control. E. shRNA-mediated depletion of *CHKα* in MDA-MD-231 cells inhibited extravasation. Control (PLKO-infected) cells and shRNA-mediated depleted CHKα MDA-MB-231 cells were injected into the blood circulation of 48 hpf zebrafish. Representative images of the zebrafish at 6 days post-injection are shown. F. Quantification of the results shown in (E). The number of cells that extravasated in the posterior tail fin was calculated at 6 dpi.

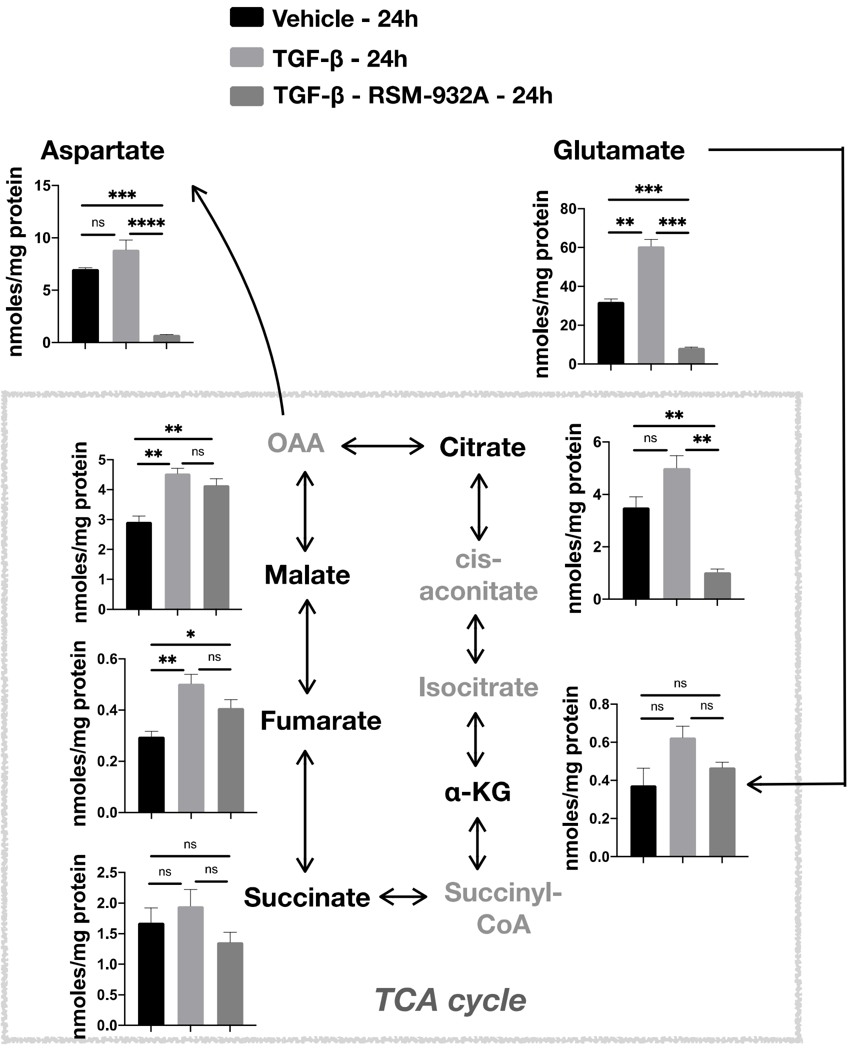

Figure S6. Effect of TGF-β in the absence and presence of RSM-932A on TCA cycle intermediates in MCF10A-Ras cells. TGF-β induced an increase in TCA cycle intermediates in MCF10A-Ras, and this was decreased by

RSM-932A (10µM). Results are shown as mean ± s.d; Results are shown as mean ± s.d; *p< 0.05; **p< 0.01; ***p<0.001; ****p< 0.0001; ns, non-significant. αKG, α-ketogluturate; TCA, Tricarboxylic acid cycle.

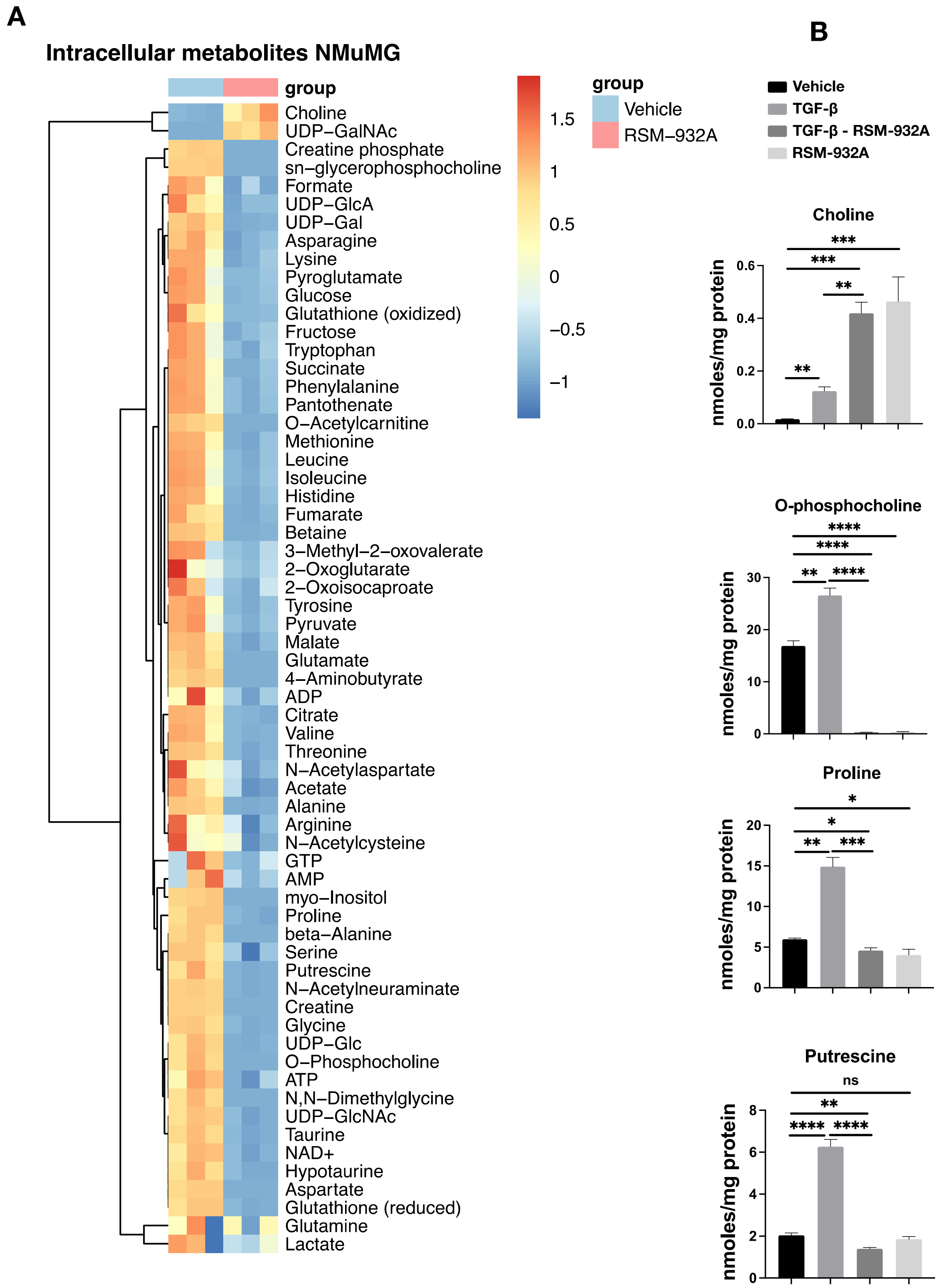

Figure S7. Analysis of RSM-932A and/or TGF-β treatment in NMuMG cells on choline metabolism. A. Analysis of RSM-932A (10μM) -induced intracellular metabolic changes in NMuMG after 24 h treatment. B. Analysis of changes in choline, O-phosphocholine, proline and putrescine levels in response to RSM-932A (10μM) and/or TGF-β (5ng/mL) treatment in NMuMG cells. Results of three biological replicates are shown as mean ± s.d; *$p<0.05$; **$p< 0.01$; ****$p< 0.0001$; ns, non-significant.

Supplementary Table S1~~:~~. List of RT-qPCR primers.

Gene		Sequence (5' to 3')
**CPT**	Forward	ACCGGAACAGGTTCAAGTTCTTT
	Reverse	TGAAACAGCAGCAGCAAGAGA
**PCYT1A**	Forward	TGACCCCCGGAGCAGTAAAT
	Reverse	CTTTTAATCGGTCATGGTCCCGT
**PCYT1B**	Forward	GAGACGAAAGAAAGTTGGGGC
	Reverse	GTCAGGGTCTTGCGTGAGAA
**CHKA**	Forward	GCCTACCTGTGGTGTAAGGAA
	Reverse	ACCACCCCTGATGACACTGA
**SLC44A1**	Forward	AAGGGAAGGAAAACGCTTGTG
	Reverse	GCTCTGCTCCAGGCTTTCAA
**SLC44A4**	Forward	AGACTGAGCCATGGGGAGAA
	Reverse	GCAGCCCCTGTTTTTGATGG
**SLC5A7**	Forward	TGCTGTCTTTTGCAACCCAG
	Reverse	TCCCAAGTCAAGTCGTCTGC
**CHAT**	Forward	AAATGGCGTCCAACGAGGAT
	Reverse	CCCGGTTGGTGGAGTCTTTT
**PEMT**	Forward	AGGCTTCGGCAATATCGACT
	Reverse	CCATCTCGCTACCACATTCCA
**ALDOA**	Forward	GGCAGTGGGAGGCAATATCT
	Reverse	GCAGTGCTTTCCGGTCTTTAG
**PGI**	Forward	TACGAACACGGCCAAAGTGA
	Reverse	AGTCCAATGGCTGACCACAG
**GLUT1**	Forward	GGGTCTTAAGTGCGTCAGGG
	Reverse	TCACCTTCTTGCTGCTGGG
**HK2**	Forward	TCGGTTTCTCTATTTGGCCCC
	Reverse	GGTCAACCTTCTGCACTTGG
**PFKFB3**	Forward	AGAGGTCAGAGAACATGAAGAGC
	Reverse	TTGGCCTCGAGAAGATGAGC
**PHGDH**	Forward	ATGTCATCAGCTGTCCCCAC
	Reverse	GCGTTCACAACGCCTGTTAG
**GAPDH**	Forward	GGCAAATTCAACGGCACAGT
	Reverse	TAGGGCCTCTCTTGCTCAGT
**LDHA**	Forward	GATGGCAACCCTCAAGGACC
	Reverse	TCATCCGCCAAGTCCTTCATT
**LDHB**	Forward	GGTGGACAGGGAATGTACGG
	Reverse	GAGCGACCTCATCGTCCTTC
**LDHC**	Forward	CGGAGTCAGCAGTAAGGCTC
	Reverse	GCCATGCCCACATTTCCAAC
**MPC1**	Forward	GACTCCCACGGTAGATGCAC
	Reverse	ACAGAGGGCGAAAGTCATCC

Supplementary Table S1. List of RT-qPCR primers (*continued*).

**MPC2**	Forward	TTGAGGCCGCTTTACAACCA
	Reverse	CAGCACACACCAATCCCCAT
**PDHA1**	Forward	AGATGCTTGCCGCTGTATCC
	Reverse	GCCGATGAAGGTCACATTTCTTAAT
**PKLR**	Forward	AAAATTGGCCCAGAGGGACT
	Reverse	CCGAAGCGCAGATCCAAAAG
**PKM1**	Forward	AGCACCTGATAGCTCGGGA
	Reverse	GAGGTCTGTGGAGTGACTGG
**LDHAL6B**	Forward	GAGAAATGGGGCGATGTCCA
	Reverse	GCCAATGGCCCAGGAAGTAT
**PDK1**	Forward	GCAGACAGCTTACCTGAATCCT
	Reverse	ACTGACCCGGTCTATAAAGTAATCC
**PDK2**	Forward	GGAGGTGCTAACAGGGGAAC
	Reverse	TGCTGGATCCTCATGGTAAGTT
**PDK3**	Forward	TAGGGAGTATCGACCCCACC
	Reverse	TTTGGCGCTTTGGCATTGAA
**PDK4**	Forward	GCTGCTGGACTTTGGTTCAGA
	Reverse	TTCAGGATATTGGCCAGGCG
**ACO2**	Forward	CATGTGGCCTCTGTCCTGTG
	Reverse	GTTCAACCGTTTACGGACAATGTTA
**MDH1**	Forward	ATGATGGGTGTTCTGGACGG
	Reverse	GTCCGTTGCAATGACATCCTG
**MDH2**	Forward	AGAGTGGAGGTCGTTGGAGT
	Reverse	CATTGTTCTGGGCCGAAGTG
**CS**	Forward	TGGGAGCCAAGAACTCATCC
	Reverse	TGTCTTCCCATGTTGCTGCT
**FH**	Forward	ATGCCAGGAAAGGTGAACCC
	Reverse	ACAGCAACGTGATTCCCCAT
**IDH1**	Forward	GCCAGTCGCTGTTACCGTAT
	Reverse	TCAATAACCCTCTTCACTCTGGAC
**IDH2**	Forward	GACAGTCACCCGCCATTACC
	Reverse	AGCGTCTGTGCAAACCTGATA
**OGDH**	Forward	CCCTGGGGATTTTGGATGCT
	Reverse	TGTAGGCCATAGAACCCTCCT
**SUCLA2**	Forward	ACGGGTCGGACTCAGAAATG
	Reverse	ACATCCTTTGATCCTGGGCG
**SUCLG1**	Forward	TACGGAAGGTATTCCGCAGC
	Reverse	TTCTCCAGGGTTGATGACGC
**GDH1**	Forward	AGGGTTTTATTGGTCCTGGCA
	Reverse	ATGGTGCTGGCATAGGTGTC
**GLS2**	Forward	CAGCACTCGGATCATGACG
	Reverse	CAGTCCAGTGGCCTTCAGAG
**GOT1**	Forward	CACCAACCTGGGAGAACCAT

Supplementary Table S1~~:–~~. List of RT-qPCR primers (*continued*).

	Reverse	CTCCGCATCCCAGTAGCAAT
**GOT2**	Forward	GGGGCCAGTTTTCTGCAAAG
	Reverse	AGATGGGCGTGTGATTTCCC
**SLC1A5**	Forward	TCGTCTTTGGTGTGGCTCTG
	Reverse	AACGGGTGCGTACCACATAA
**SLC7A5**	Forward	CTGGTCTTCGCCACCTACTT
	Reverse	GCCTTTACGCTGTAGCAGTTC
**GLS1**	Forward	CCGCGGGCGACAATAAAATA
	Reverse	GGGATCAGATGTTCGCAATCC
**6PGDH**	Forward	CTCCTCGACTCTGCTTCGTC
	Reverse	TGAAAGCACAGACCACAAATCC
**6PGL**	Forward	ACTCAAGATACAAGGGCCGC
	Reverse	CGTTGAAGCAGATCCTTGCG
**G6PD2**	Forward	GGTCGTGGGGGCTATTTTGA
	Reverse	TCTGAATCTGTGGTAGCAGGC
**G6PDX**	Forward	GAACGGTGGGACGGGGTA
	Reverse	CTGCCACGTCTCGGAACTG
**RPE**	Forward	CCTGGATGCACCCTGCTC
	Reverse	CTAGCTGCTTTCGGAGGCTT
**RPI**	Forward	CCCAGAGATTGACCTTGCCA
	Reverse	ACTTGCATAACCAGCCACGA
**TALDO**	Forward	CAACGAAGACCAAATGGCCG
	Reverse	CATTCGTTCCGTGAGCATCC
**TKT**	Forward	TGTCCCGAAACAAGCCTTCA
	Reverse	ACTCGGTAGCTGGCTTTGTC
**GNPNAT1**	Forward	CCGCTCCAGTGCGACTTTA
	Reverse	GGATGCGTTGGAGAAATGGC
**OGT**	Forward	AGCTATCGAACTGCAACCCC
	Reverse	TTCAGCAACACTGCCCTTCT
**PGM3**	Forward	TTATTCAACCAGGTCGCGGA
	Reverse	AGAGGGCCGTACAAAAGCTC
**GFPT1**	Forward	TCTGATGCAAGTGCCGTGAT
	Reverse	GATGGTCTCCTGCAGTTCGT
**GFPT2**	Forward	ATCCTAGCCGGAGAGCTGAA
	Reverse	GTATGATTGGGCGACCCTGG
**UAP1**	Forward	CATCCCCCGCAGTGCTAC
	Reverse	AGGCCCTCTCCAGCATAAGA
**HAS2**	Forward	CGTGGATTATGTACAGGTGTGTG
	Reverse	TCCTCCAACACCTCCAACCA
**B3GNT9**	Forward	CGTCACCCTGTCCAGACTTTG
	Reverse	CATCCACTCTGTCGACCTCC
**BCAT1-1**	Forward	ACCCGCCACGCCAAAC
	Reverse	TTACTGCAGTCCTGGCGG

Supplementary Table S1~~: –~~. List of RT-qPCR primers (*continued*).

**BCAT1-2**	Forward	GATACAAATCACTGGAGGGG
	Reverse	GGAAGATGGAGAGCTGGG
**BCAT2**	Forward	TATGGACCCACTGTGGCTGT
	Reverse	AGCTCCAGTACTCCGTCTTCA
**BHMT**	Forward	GAGGCAGTTCGTCAGCTTCA
	Reverse	TCGGGGTTCCAATCCAAAGG
**MTHFD1**	Forward	CTGACCCAGACAGACAAGGC
	Reverse	ATGCCTAGCCTCCGTAACCT
**MTHFD1L**	Forward	TTTGGTGGGAACGATGAGCA
	Reverse	CCAGCCTTGACCGCATCTT
**MTHFR**	Forward	GAGCAGATCGGGATGAACCA
	Reverse	GATTCCTGCTGATAGAGGGTGG
**MTR**	Forward	AGGAATACTTGCTGGCTGGAG
	Reverse	GTTCATCCGGTAGGCCAAGT
**SHMT1**	Forward	CCACGCTCCTAATACAAGGCA
	Reverse	TGCTGTAAACCTCGGCATCA
**SHMT2**	Forward	ACCCCGGTACTACACCGATA
	Reverse	AGCTGACCACATCTCCGAGT
**DHFR**	Forward	AGGATTTTATCCCCGCTGCC
	Reverse	GTACTTGAACTCGTTCCTGAGC
**CA9**	Forward	AAGACAGGATGGAGGAGTCCC
	Reverse	TGGCCACCCCCTTTTTCATC
**CA12**	Forward	TGGATGATCCTACCCCCAGA
	Reverse	GGATGATACCTTGTCGGAAGGAG
**ODC**	Forward	TCCCTGTCACATCGAGAACC
	Reverse	CCGCAACATAGAACGCATCC
**DDAH1**	Forward	TGCGTGTTCGTGGAGGAC
	Reverse	ATGTCAACCTCCTTCCTGCG
**MICAL2**	Forward	CCCCCAGTACGTTTCAGCC
	Reverse	TTGCCAAGAAAATCAGTCACCTT
**PLPP3**	Forward	CCTGGCGATCATTACAGGGG
	Reverse	TGCCACATACGGGTTCTGAG
**PLPP4**	Forward	ACCTACCCGCCTCATGTTTG
	Reverse	GAGCCAAGGACACTGCTAAGA
**PLCB4**	Forward	AAAGAGTTCCGAACCCGCAT
	Reverse	TCAGGCAGGATCACCTTTCG
**SULF1**	Forward	ATTAAGAGAAGGAAGGCTGCCC
	Reverse	CTCAGCACCTGAAAATACTGCG
**GPX8**	Forward	AAGGCAAAGCTTCCCTGGTT
	Reverse	GATAGGGCCCGAACTCCTTG
**SPHK1**	Forward	TCCTGGGCAACACCGATAAG
	Reverse	CGAGGGCATTCTACTGGTTCC
**AOX1**	Forward	AGAACGGAAGCTGGAGTGTG

Supplementary Table S1:. List of RT-qPCR primers (*continued*).

	Reverse	CCGCCTATGTGTATTTCACCTTC
**CYP1B1**	Forward	TGTGCCTGCCACTATTACGG
	Reverse	CTGAACATCCGGGTATCTGGT

Supplementary Table S2: Changes in extracellular metabolites in the absence or presence of TGF-β or RSM-932A, and TGF-β-induced in intracellular metabolites in MCF10A-Ras cells. This Table is provided in an excel sheet format.

Supplementary Table S3: TGF-β-induced kinetic effects on gene expression encoding metabolic enzymes in NMuMG cells. This Table is provided in an excel sheet format.
